# Multilayer Perceptron for Robust Nonlinear Interval Regression Analysis Using Genetic Algorithms

**DOI:** 10.1155/2014/970931

**Published:** 2014-06-29

**Authors:** Yi-Chung Hu

**Affiliations:** Department of Business Administration, Chung Yuan Christian University, Chung Li 32023, Taiwan

## Abstract

On the basis of fuzzy regression, computational models in intelligence such as neural networks have the capability to be applied to nonlinear interval regression analysis for dealing with uncertain and imprecise data. When training data are not contaminated by outliers, computational models perform well by including almost all given training data in the data interval. Nevertheless, since training data are often corrupted by outliers, robust learning algorithms employed to resist outliers for interval regression analysis have been an interesting area of research. Several approaches involving computational intelligence are effective for resisting outliers, but the required parameters for these approaches are related to whether the collected data contain outliers or not. Since it seems difficult to prespecify the degree of contamination beforehand, this paper uses multilayer perceptron to construct the robust nonlinear interval regression model using the genetic algorithm. Outliers beyond or beneath the data interval will impose slight effect on the determination of data interval. Simulation results demonstrate that the proposed method performs well for contaminated datasets.

## 1. Introduction

In many practical applications, since the available information is often derived from uncertain assessments, real intervals can be employed to represent uncertain and imprecise observations [1]. Interval regression analysis, which provides interval estimation of individual dependent variables, is an important tool for dealing with uncertain data [1–3]. Interval regression analysis was developed on the basis of an important tool, namely, fuzzy regression analysis introduced by Tanaka et al. [4], whose objective is to build a model that contains all observed output data in terms of fuzzy numbers [4, 5].

Among computational models in intelligence, neural-network-based approaches have been employed to deal with nonlinear fuzzy or interval regression analysis (e.g., [6–10]) to facilitate the usefulness of fuzzy regression analysis. It is known that the neural-network-based approaches can overcome the difficulty in nonlinear fuzzy regression with LP, which is to choose a nonlinear model from an infinite number of alternatives [2]. When training data are not contaminated by outliers, which can be simply interpreted as data with a large deviation from its designated location [3, 11], these methods perform well and the estimated data interval includes almost all given training data in the data interval. Nevertheless, since training data are often corrupted by outliers, data interval obtained by these methods can be influenced by outliers. It is noted that statistical data preprocessing is helpful to detect outliers, but the robust interval regression analysis can not only resist outliers but also provide interval estimation of individual dependent variables.

To overcome the corruption arising from outliers, robust learning approaches involving computational intelligence (e.g., neural networks and support vector machines) have been employed to determine the upper and lower bounds of data interval. For instance, Huang et al. [3] employed two MLPs to determine nonlinear interval models using a new cost function, in which the cost function introduced in [6] and the robust BP algorithm for function approximation [12] were taken into account. Jeng et al. [2] employed two radial basis function networks to determine the upper and lower bounds after applying the support vector regression approach to determine the initial structure and parameter values of neural networks. Moreover, Hwang et al. [1] proposed a robust method by combining the possibility estimation formulation integrating the property of central tendency with the principle of support vector interval regression.

It is found that, to determine the maximum and minimum limitations of interval regression, Jeng et al. [2] estimated the trimmed standard deviation of residuals excluding the highest and lowest *α*% of the number of training data. *α* can be specified as zero and 5–10 without and with outliers contained in the data, respectively. To avoid the estimated upper and lower bounds going beyond the maximum and minimum limitations, the values of a constant in the robust learning algorithms, introduced in [1, 2], are required to be carefully specified for both the uncontaminated and the contaminated data, to modify the desired output of each pattern. Moreover, in [3, 13], the upper limits on the percentage of outliers beyond and beneath the true interval model are prespecified for determining the degree of influence of each training pattern. Although the above-mentioned robust learning algorithms are robust against outliers, the required parameters for these algorithms are related to whether the collected data contain outliers or not. However, since the collected data are more or less contaminated, it is not easy to prespecify the degree of contamination. This motivates us to develop robust method without considering the degree of contamination.

On the basis of the effectiveness of using two MLPs to independently identify the upper and lower bounds of data interval for interval regression analysis, this paper aims to propose robust learning algorithms with the weighting schemes in [6] for MLP by using the genetic algorithm (GA) to identify outliers automatically for determining the robust nonlinear interval regression model, since the GA is a powerful search and optimization method [14, 15]. The proposed method has the feature that outliers beyond or beneath the data interval will impose slight effect on the determination of upper and lower bounds. In practice, only parameter specifications for the GA are required for the proposed learning approach. To sum up, the main contribution of this paper is to use MLP to construct a robust nonlinear interval model by making use of the GA.

The rest of this paper is organized as follows. The functional-link net with functional-expansion model for approximation is introduced in Section 2.  Section 3 introduces the MLP-based approach proposed by Ishibuchi and Tanaka [6] for nonlinear interval regression analysis.  Section 3 describes the proposed robust learning algorithms in detail. In Section 4, in order to examine the effectiveness and applicability of the proposed method for determining a nonlinear interval regression model, several examples and real data are taken into account. From the experimental results, it is seen that the nonlinear interval models obtained by the proposed learning algorithms can include almost all regular data. This paper is concluded in Section 5.

## 2. Multilayer Perceptron for Nonlinear Interval Regression Analysis

Since the weighting schemes proposed by Ishibuchi and Tanaka [6] are incorporated into the proposed robust learning algorithms, the MLP-based approach employed to determine the estimated data interval is described in this section. MLP and the MLP-based approach are briefly reviewed in Sections 2.1 and 2.2, respectively.

### 2.1. Multilayer Perceptron

MLP is usually used as a tool of approximation of functions like regression [16]. A three-layer perceptron with *n* input nodes and a single hidden layer is taken into account. Let us denote the given nonfuzzy input-output pairs by (**x**_*p*_, *y*_*p*_), *p* = 1, 2,…, *m*, where **x**_*p*_ = (*x*_*p*1_, *x*_*p*2_,…, *x*_*pn*_) and *y*_*p*_ are the input vector and the corresponding desired output value, respectively. The sigmoid function, whose output ranges from 0 to 1, is commonly used as the transfer function for each hidden node and output node.

When **x**_*p*_ is presented to MLP, the output value from the *j*th hidden node is computed as

(1) $$\begin{array}{c} o_{pj}=f_{s}\left(\overset{n}{\underset{i=1}{\sum }}w_{ij}x_{pi}+\theta_{j}\right), \end{array}$$

where *f*_*s*_ represents the sigmoid function, *θ*_*j*_ is the bias to the *j*th hidden node, and *w*_*ij*_ is the connection weight from the *i*th input node to the *j*th hidden node. Then, the final output value from the output node is computed as

(2) $$\begin{array}{c} o_{p}=f_{s}\left(\overset{k}{\underset{j=1}{\sum }}w_{j}o_{pj}+\theta \right), \end{array}$$

where *k* is the number of hidden nodes, *θ* is the bias to the output node, and *w*_*j*_ is the connection weight from the *j*th hidden node to the output node. Thus, there are (*n* + 2)*k* + 1 synaptic connections. The following cost function can be employed to train MLP:

(3) $$\begin{array}{c} E=\frac{1}{2}\overset{m}{\underset{i=1}{\sum }}{\left(y_{p}-o_{p}\right)}^{2}, \end{array}$$

where *m* represents the number of training patterns. It should be noted that since it was shown that MLP with a single hidden layer and any fixed continuous sigmoid function is sufficient to approximate any continuous function [17], a three-layer model is taken into account for our study.

### 2.2. MLP-Based  Approach

Ishibuchi and Tanaka [6] employed two feed-forward MLPs, MLP* and MLP_∗_, to determine effectively a nonlinear interval model. Each of the two networks has only one hidden layer. Let g*(**x**) and *g*_∗_(**x**) denote the output functions realized by MLP* and MLP_∗_, respectively. In practice, g*(**x**) and *g*_∗_(**x**) represent the upper and lower bounds of a nonlinear interval model, respectively.

A nonlinear optimization problem is formulated to determine the nonlinear interval regression model as follows:

(4) Minimize (g∗(x1)−g∗(x1))+(g∗(x2)−g∗(x2))+⋯+(g∗(xm)−g∗(xm))subject  to g∗(xp)≤yp≤g∗(xp), p=1,2,…,m,

where (g*(**x**_*p*_) − *g*_∗_(**x**_*p*_)) represents the width of the estimated data interval for **x**_*p*_. The objective of the above formulation is to determine the nonlinear interval model with the least sum of widths of the predicted intervals for the respective inputs subject to the estimated data interval determined by the two MLPs including all the given input-output pairs.

Instead of deriving a learning algorithm directly from the above nonlinear optimization problem, Ishibuchi and Tanaka derived learning algorithms for g*(**x**) and *g*_∗_(**x**) by the following cost function with weighting scheme *ω*_*p*_:

(5) $$\begin{array}{c} E=\overset{m}{\underset{p=1}{\sum }}\frac{1}{2}\omega_{p}{\left(y_{p}-g^{*}\left(x_{p}\right)\right)}^{2}, \end{array}$$

where *ω*_*p*_ is a small positive value in the interval (0, 1). To determine the upper bound g*(**x**), *ω*_*p*_ is defined as

(6) $$\begin{array}{c} \omega_{p}=\{\begin{array}{cc} 1, & \text{if}\;\;y_{p}>g^{*}\left(x_{p}\right),\\ \omega , & \text{if}\;\;y_{p}\leq g^{*}\left(x_{p}\right). \end{array} \end{array}$$

As for determining the lower bound *g*_∗_(**x**), *ω*_*p*_ is defined as

(7) $$\begin{array}{c} \omega_{p}=\{\begin{array}{cc} 1, & \text{if}\;\;y_{p}<g_{*}\left(x_{p}\right),\\ \omega , & \text{if}\;\;y_{p}\geq g_{*}\left(x_{p}\right). \end{array} \end{array}$$

It can be seen that the cost function can be multiplied by a small positive value in the interval (0, 1) (i.e., *ω*_*p*_) depending on whether the desired output of an input pattern is less than or greater than the network-estimated value. Weights updating rules in the MLP-based approach can be derived by gradient descent from the above objective function with weighting scheme. It is obvious that the two learning algorithms for determining g*(**x**) and *g*_∗_(**x**) are the same except for the weighting schemes. Ishibuchi and Tanaka suggested that a smaller value of *ω*_*p*_^(*r*)^ could lead to better satisfaction of the constraint condition.

Nevertheless, the nonlinear interval model derived by the MLP-based approach can include all training data with outliers [3]. That is, the nonlinear interval model obtained by the MLP-based approach is sensitive to contaminated training data. To overcome the above problem, Huang et al. [3] pointed out that we should only pay attention to the quality of the training data whose desired outputs are greater than the actual outputs for determining g*(**x**) and that of the training data whose desired outputs are less than the actual output for determining *g*_∗_(**x**). In practice, those more suspected outliers beyond or beneath the estimated data interval impose slighter effect on the determination of data interval.

## 3. The Proposed Robust Learning Algorithms

According to the objective function with the weighting schemes introduced in [6], Section 3.1 demonstrates the formulation of optimization problems. Moreover, the fitness functions of the GA are described. Subsequently, the coding scheme and genetic operations are described in detail in Sections 3.2 and 3.3, respectively. Finally, the proposed robust learning algorithms for determining the nonlinear interval model are presented.

### 3.1. Problem Formulation

Two neural networks, MLP* and MLP_∗_, are still employed to determine the upper and lower bounds of data interval. To determine the upper bound of the nonlinear interval regression model (i.e., g*(**x**)), the single-objective optimization problem of constructing MLP* is formulated as

(8) Minimize E(MLP)subject  to yp≤g∗(xp), p=1,2,…,m.

In a similar manner, the single-objective optimization problem of constructing MLP_∗_ for the lower bound (i.e., *g*_∗_(**x**)) is formulated as

(9) Minimize E(MLP)subject  to g∗(xp)≤yp, p=1,2,…,m,

where *E*(MLP) is defined as

(10) $$\begin{array}{c} E\left(\text{MLP}\right)=\overset{m}{\underset{p=1}{\sum }}\frac{1}{2}\omega_{p}\Psi_{p}{\left(y_{p}-f^{*}\left(x_{p}\right)\right)}^{2}. \end{array}$$

It can be seen that Ψ_*p*_ is incorporated into the above-mentioned cost function *E*. In the determination of g*(**x**) at the *t*th generation during evolution, Ψ_*p*_ for **x**_*p*_ is set to be of a very small positive value, say Ψ, if **x**_*p*_ beyond g*(**x**) is an outlier. On the contrary, Ψ_*p*_ is set to be 1. In a similar manner for determining *g*_∗_(**x**), Ψ_*p*_ for **x**_*p*_ is set to be of a very small positive value if **x**_*p*_ beneath g*(**x**) is an outlier. Otherwise, Ψ_*p*_ is set to be 1. In other words, outliers beyond or beneath the data interval can impose a slight effect on the determination of data interval.

The single-objective GA, which is a general-purpose optimization technique, can be applied to the above problems by introducing a reward to the fitness function when the constraint condition is satisfied:

(11) $$\begin{array}{c} \text{fitness}\left(\text{MLP}\right)=w_{e}\cdot \frac{1}{1+E\left(\text{MLP}\right)}+w_{\text{num}}\cdot R\left(\text{MLP}\right), \end{array}$$

where *w*_*e*_ and *w*_num_ are constant positive weights of the objective and reward, respectively, and *R*(MLP) is the ratio of input-output pairs that satisfy the constraint condition:

(12) $$\begin{array}{c} R\left(\text{MLP}\right)=\frac{1}{m}\overset{m}{\underset{p=1}{\sum }}r_{p}, \end{array}$$

where *r*_*p*_ is defined for determining g*(**x**) as

(13) $$\begin{array}{c} r_{p}=\{\begin{array}{cc} 0, & \text{if}{\;\;y}_{p}>g^{*}\left(x_{p}\right),\\ 1, & \text{if}{\;\;y}_{p}\leq g^{*}\left(x_{p}\right), \end{array} \end{array}$$

whereas for determining *g*_∗_(**x**), *r*_*p*_ is defined as

(14) $$\begin{array}{c} r_{p}=\{\begin{array}{cc} 1, & \text{if}{\;\;y}_{p}>g_{*}\left(x_{p}\right),\\ 0, & \text{if}{\;\;y}_{p}\leq g_{*}\left(x_{p}\right). \end{array} \end{array}$$

The reward *R*(MLP) is incorporated into the fitness function (i.e., fitness(MLP)) in order to facilitate all regular data beyond or beneath the estimated data interval.

### 3.2. Coding

As in [13], the transformation of the network output with respect to the *p*th pattern, say *o*_*p*_, to the domain interval of the desired output is performed as follows:

(15) $$\begin{array}{c} o_{p}=o_{p}\left(ma-mi\right)+mi,\;p=1,2,\ldots ,m, \end{array}$$

where *ma* and *mi* equal (max⁡ {*y*_*p*_∣*p* = 1, 2,…, *m*} + *u*) and (min⁡ {*y*_*p*_∣*p* = 1, 2,…, *m*} − *l*), respectively. Those unknown parameters including *u*, *l*, (*n* + 2)*k* + 1 connection weights and bias of a MLP are automatically determined by the GA. Thus, there are (*n* + 2)*k* + 3 substrings in a binary string. To identify outliers, a substring consisting of *m* bits is employed to determine the set of outliers, and each bit can indicate whether the corresponding training pattern is an outlier or not. In practice, the bit of 0 indicates that the corresponding training pattern is not an outlier; otherwise, that pattern is an outlier. In an initial population, each bit in the string is randomly assigned as either 1 or 0, with the probability of 0.5. A substring except for determining outliers and *u*, *l* can be directly decoded as a real value ranging from −5 to 5. Such a range should be acceptable when the sigmoid function is used as the activation function, since it is known that a node tends to suffer from the premature saturation resulting from infinitely large positive or negative weights.

The determination of the length of a string depends mainly on their domain lengths and the corresponding required precision [18]. That is, if the domain of a variable has the length of *τ*_1_ where 2^*τ*_3_−1^ < *τ*_1_10^*τ*_2_^ < 2^*τ*_3_^, and the corresponding required precision is *τ*_2_ decimal places, then *τ*_3_ bits are required to code such a variable.

### 3.3. Genetic Operations

Let *N*_pop_ denote the population size. When the fitness of each chromosome in the current population is obtained, genetic operators including selection, crossover, and mutation [14, 15, 19] are employed to determine the newly generated *N*_pop_ strings in the next population. Using the binary tournament selection with replacement, two strings are randomly selected from the current population, and the one with the maximum fitness can be placed in the mating pool. This process can be repeated *N*_pop_ times until there are *N*_pop_ strings in the mating pool. In other words, 0.5*N*_pop_ pairs of chromosomes can be randomly selected from the current population.

After tournament selection, crossover and mutation are applied to a selected parent to reproduce children by altering the chromosomal makeup of two parents. In practice, the one-point crossover operation with the crossover probability Pr_*c*_ is used for exchanging partial information between two substrings in the selected pair of strings, and two new strings are generated to replace their parent strings. Each crossover point in a substring is chosen randomly. That is, we use a substring-wise one-point crossover operation where the total number of crossover points is the same as the number of substrings in each string. The mutation operation with the mutation probability Pr_*m*_ is performed on each bit of strings generated by the crossover operation.

### 3.4. Learning Algorithm Implementation

The MLP robust learning algorithm for determining the upper bound of the nonlinear interval regression model (i.e., g*(**x**)) is the same as that for determining the lower bound (i.e., *g*_∗_(**x**)), except for the weighting scheme, *R*(MLP), and Ψ_*p*_ in the fitness function. The learning of each of the two MLPs is independent of each other. That is, two MLPs are independently employed to determine the upper and lower bounds of data interval. The learning algorithm for determining g*(**x**) is written in the following.

*Algorithm 1* (a robust MLP learning algorithm for determining the upper bound of a nonlinear interval model).

*Input*

- Population size: *N*_pop_;
- Total number of generations: *N*_con_;
- Number of elite chromosomes: *N*_del_;
- Crossover rate: Pr_*c*_;
- Mutation rate: Pr_*m*_;
- Relative weights in the fitness function: *w*_*e*_ and *w*_num_;
- A set of training patterns.

*Output.* The upper bound of a nonlinear interval model.

*Method*

*Step 1* *: Initialization*. A population containing *N*_pop_ binary strings is generated randomly.

*Step 2* *: Transform Network-Estimated Values*. For each pattern, transform the output of MLP into a value ranging from (max⁡{*y*_*p*_∣*p* = 1, 2,…, *m*} + *u*) to (min⁡{*y*_*p*_∣*p* = 1, 2,…, *m*} − *l*) by (15).

*Step 3* *: Compute Fitness Values. *Compute the fitness value of each string in the current population by (11).

*Step 4* *: Termination Test*. *N*_con_ is used as the stopping condition. If the stopping condition is not satisfied, then proceed to the next step. That is, the genetic operations are iterated again to generate the new strings in the next population.

*Step 5* *: Generate New Strings*. Genetic operators are employed to generate the *N*_pop_ new strings in the next population from the current population.

*Step 6* *: Perform Elitist Strategy*. *N*_del_ strings are randomly removed from the newly generated *N*_pop_ strings. Then, add *N*_del_ best strings in the current population to form the next one.

The best string among the successive generations is taken as the desired solution.

## 4. Computer Simulations

Several data sets are employed to examine the effectiveness of the proposed method for determining a nonlinear interval regression model. Each data set is involved in learning a function of one variable (i.e., *n* = 1). Since there is no best set of GA parameter specifications, according to the principles introduced in [18], the prespecified parameter specifications of the proposed robust learning algorithms for each simulation are described as follows.

1. *N*_pop_ = 50: the most common population size varies from 50 to 500 individuals. Hence, 50 individuals is an acceptable minimum size. In an initial population, each bit in a binary string is randomly assigned as either 1 or 0, with the probability of 0.5.
2. *N*_con_ = 5000: the stopping condition is specified according to the available computation time. However, a sufficient evolution of the GA is necessary.
3. *N*_del_ = 2: to avoid generating too much perturbation in the next generation, a small number of elite chromosomes are taken into account.
4. Substring length: (i) since a connection weight ranges from −5 to 5 (i.e., *τ*_1_ = 10), and the required precision is three decimal places (i.e., *τ*_2_ = 3), 14 bits (i.e., *τ*_3_ = 14) are required to code each connection weight. (ii) Both *u* and *l* are set to be small real values in [0, 3] (i.e., *τ*_1_ = 5), and the required precision is also three decimal places. Therefore, 13 bits are required to code both *u* and *l*.
5. Pr_*c*_ = 0.95 and Pr_*m*_ = 0.001: since a Pr_*c*_ with a larger value allows the exploration of more of the solution space, a larger Pr_*c*_ is usually taken into account. Furthermore, in order not to generate excessive perturbation, Pr_*m*_ should be specified as a lower value.
6. *w*_*e*_ = 2, *w*_num_ = 1: it is considered that the minimization of the cost function is the primary objective of the regression analysis; a larger value is thus set to be *w*_*e*_.
7. *ω* = 0.2, Ψ = 10^−5^: as mentioned above, *ω* is suggested to be specified as a small value. Ψ is set to be a very small positive value approaching zero.

Besides, as in [6], a MLP with five hidden nodes (i.e., *k* = 5) is taken into account. Therefore, there are 18 substrings in a binary string.

First, uncontaminated data are employed to verify the effectiveness of the proposed learning algorithms. The uncontaminated training data of the first example generated by

**Figure 1:**
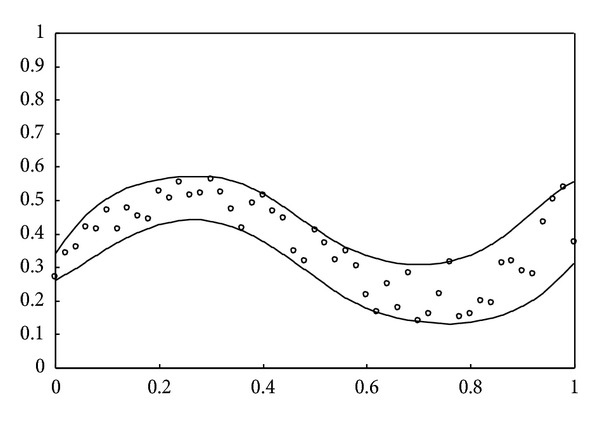
Simulation result for the first example without outliers.

(16) xp=0.02(p−1), p=1,2,…,51,yp=0.2sin(2πxp)+0.2xp2+0.3 +(0.1xp2+0.05)rnd[−1,1]

are taken into account, where rnd[−1,1] denotes a real number generated in the interval [−1, 1] at random. The simulation result obtained using the proposed learning algorithms is depicted in Figure 1. As can be seen, 51 training data are approximately included in the data interval. For the second example, a simple function for generating the uncontaminated training data is defined as

(17) $$\begin{array}{c} x_{p}=0.08\left(p-1\right)-2,\;p=1,2,\ldots ,51,\\ y_{p}=\exp\left(-x_{p}^{2}\right)+0.5\text{rnd}\left[-1,1\right]. \end{array}$$

**Figure 2:**
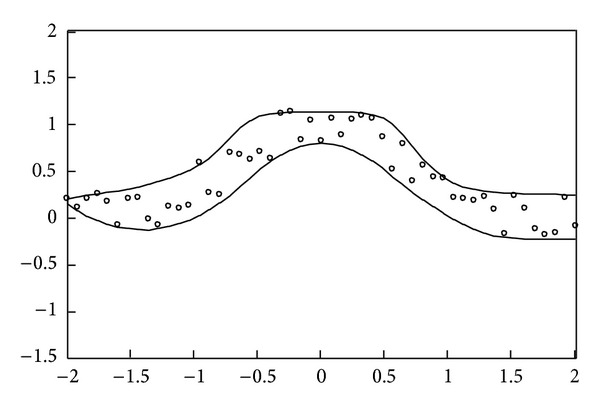
Simulation result for the second example without outliers.

**Figure 3:**
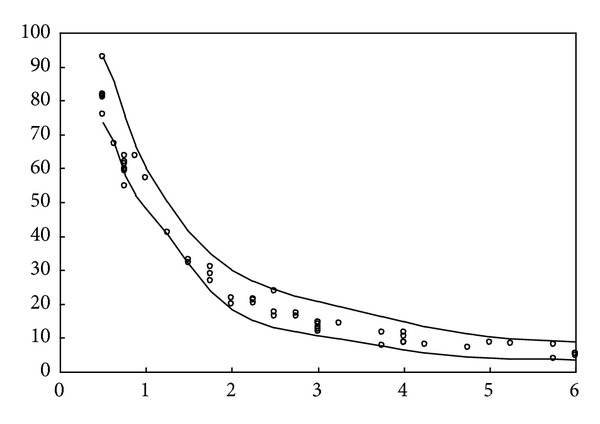
Simulation result for the ultrasonic calibration data.

**Figure 4:**
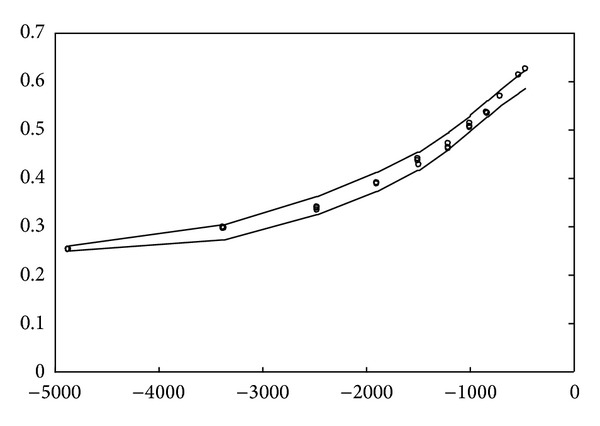
Simulation result for the data of quantum defects in iodine atoms.

The simulation result obtained using the proposed method for the above uncontaminated data is depicted in Figure 2. Moreover, as can be seen, the data interval includes almost all given training data. Subsequently, the simulation results for the real data set of studies on National Institute of Standards and Technology (NIST), which involve ultrasonic calibration consisting of 54 observations [20] and quantum defects in iodine atoms consisting of 25 observations [21], are further shown in Figures 3 and 4, respectively. The ultrasonic calibration data, whose response variable is ultrasonic response and whose predictor variable is metal distance, are often used to illustrate the construction of a nonlinear regression model. The quantum defects in data of iodine atoms, whose response variable is the number of quantum defects and whose predictor variable is the excited energy state, can also be employed to construct a nonlinear least squares regression model. It can be seen that the proposed learning algorithms work well for these two real data sets.

**Figure 5:**
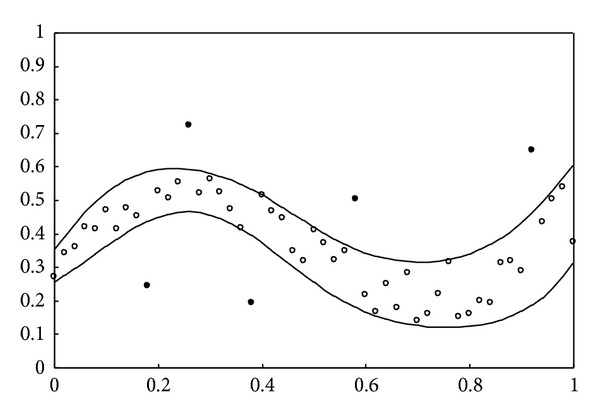
Simulation result for the first example with outliers.

**Figure 6:**
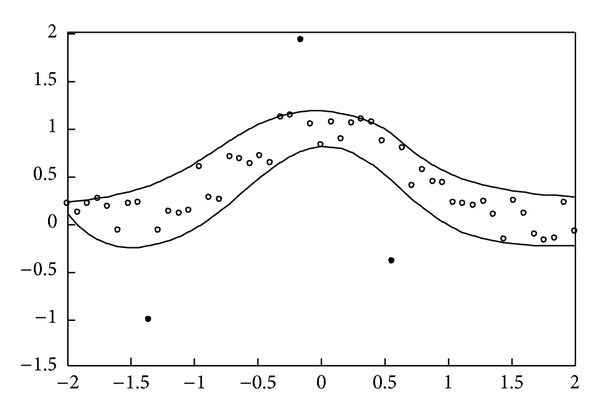
Simulation result for the second example with outliers.

Contaminated data are further employed to examine the data intervals obtained by the proposed learning algorithms. For the first example, 5 out of the same 51 input-output pairs (i.e., 46 pieces of regular data) are randomly selected as outliers. The simulation result shown in Figure 5 indicates that the proposed learning algorithms can resist outliers. Those regular data are approximately included in the robust nonlinear interval model. Furthermore, comparison with Figure 1 shows that both results are approximately identical. For the second example, 3 out of the same 51 input-output pairs are randomly selected as outliers.  Figure 6 shows that the estimated upper and lower bounds obtained by the proposed learning algorithms are not influenced by outliers. It can be seen that the simulation results shown in Figures 2 and 6 are similar. Using the traditional MLP-based approach, the results shown in [3] are not depicted in Figures 5 and 6 to simplify the presentation. From [3], it can be seen that the nonlinear interval model obtained by the MLP-based approach is sensitive to contaminated training data.

## 5. Conclusions

As mentioned above, since the available information is often derived from uncertain assessments, it is reasonable to use real intervals to deal with imprecise observations. Except for the traditional regression function determined merely by minimizing the least squared error, computational models in intelligence have been employed to determine the nonlinear interval regression model. There is no doubt that since the available data often contain outliers, the development of robust algorithms is necessary. Since the collected data are more or less contaminated, it is not easy to estimate the degree of contamination without performing statistical data preprocessing. In comparison with computational models in intelligence presented in [1–3, 13], the proposed robust learning algorithms have the advantage of avoiding considering the degree of contamination of the collected data.

This paper proposes MLP learning algorithms with the weighting schemes in [6] for determining the robust nonlinear interval regression model. Outliers, which are identified by the GA, beyond or beneath the data interval will impose a slight effect on the determination of data interval. As seen from the experimental results, it is seen that the nonlinear interval models obtained by the proposed learning algorithms can include almost all regular data. That is, the proposed learning algorithms are robust against outliers for contaminated data. Thus, it seems that the incorporation of the ratio of training data that are included in the interval model into the fitness function can facilitate the inclusion of regular data in the robust nonlinear interval model.

The nonlinear interval models shown in the previous section are satisfactory for both uncontaminated and contaminated data, whereas customized parameter tuning in computer simulations is not particularly considered for the proposed learning algorithms. The same parameter specifications in the GA are applied to each experiment. For this, it seems that the proposed learning algorithms are not sensitive to GA parameter specifications. The experimental results show that common setting of the GA parameter specifications for the proposed approach is acceptable.

Previously, several literatures with respect to the robust interval regression model have been published. For instance, Fagundes et al. [22] dealt with cases that have interval-valued outliers in the input data set; Chuang and Lee [23] used data preprocessing to filter out outliers in the training data and then a regression model could be constructed by using the filtered data to train the support vector regression networks; D'Urso et al. [24] proposed a robust fuzzy linear regression model based on the least median squares-weighted least squares estimation procedure for the highly skewed data; Huang [25] proposed a reduced support vector machine approach in evaluating interval regression models with nonfuzzy inputs and interval output, but the models seem not to pay more attention to outlier resistance. In comparison with these methods, the proposed approach uses MLP to construct the robust interval regression models with crisp inputs and crisp outputs by using the GA elaborately. The use of data preprocessing to detect outliers and the consideration of interval-valued data set remain to be studied in future work.
